# Deep breathing heart rate variability is associated with respiratory muscle weakness in patients with chronic obstructive pulmonary disease

**DOI:** 10.1590/S1807-59322010000400004

**Published:** 2010-04

**Authors:** Michel Silva Reis, Ross Arena, Ana Paula Deus, Rodrigo Polaquini Simões, Aparecida Maria Catai, Audrey Borghi-Silva

**Affiliations:** I Cardiopulmonary Physiotherapy Laboratory, Nucleus of Research in Physical Exercise, Federal University of São Carlos - São Carlos/SP, Brazil; II Departments of Internal Medicine, Physiology and Physical Therapy, Virginia Commonwealth University - Richmond, VA, USA., Tel.: +55 16 3351.9577, Email: michelsilre@hotmail.com

**Keywords:** COPD, Heart-rate variability, Respiratory sinusal arrhythmia, Respiratory muscle strength

## Abstract

**BACKGROUND:**

A synchronism exists between the respiratory and cardiac cycles. However, the influence of the inspiratory muscle weakness in chronic obstructive pulmonary disease (COPD) on cardiac autonomic control is unknown. The purpose of the present investigation was to evaluate the influence of respiratory muscle strength on autonomic control in these patients.

**METHODS:**

Ten chronic obstructive pulmonary disease patients (69±9 years; FEV_1_/FVC 59±12% and FEV_1_ 41±11% predicted) and nine age-matched healthy volunteers (64±5 years) participated in this study. Heart-rate variability (HRV) was obtained at rest and during respiratory sinusal arrhythmia maneuver (RSA-M) by electrocardiograph.

**RESULTS:**

Chronic obstructive pulmonary disease patients demonstrated impaired cardiac autonomic modulation at rest and during RSA-M when compared with healthy subjects (p<0.05). Moreover, significant and positive correlations between maximal inspiratory pressure (MIP) and the inspiratory-expiratory difference (ΔIE) (r = 0.60, p<0.01) were found.

**CONCLUSION:**

Patients with chronic obstructive pulmonary disease presented impaired sympathetic-vagal balance at rest. In addition, cardiac autonomic control of heart rate was associated with inspiratory muscle weakness in chronic obstructive pulmonary disease. Based on this evidence, future research applications of respiratory muscle training may bring to light a potentially valuable target for rehabilitation.

## INTRODUCTION

The maximal inspiratory pressure (MIP) generated by the inspiratory musculature is an index of strength that can be diminished in chronic obstructive pulmonary disease (COPD)^1^ and is an independent determinant of survival in these patient populations.^2^ Traditionally, inspiratory muscle weakness in COPD has been ascribed to factors that include under-perfusion,^3^ disuse, medication, nutritional depletion, systemic inflammation,^4^ increased resistive and elastic loads, and large inequalities in time constants between different areas of the lung.^5^–^6^ A critical factor leading to impaired respiratory muscle function in patients with COPD is pulmonary hyperinflation. The shape and geometry of the chest wall is altered in these patients, leading to a chronic reduction of the diaphragmatic apposition zone.^7^

Alterations of the inspiratory muscle length-tension relationship in patients with COPD increase the sympathetic vasoconstrictor activity of the cardiovascular adjustment center in the bulb secondary to an altered ergoreflex response (muscle spindles and Golgi bodies) in the diaphragm.^7^ In this context, heart-rate (HR) autonomic control can be assessed using HR and R-R interval indexes obtained during the respiratory sinus arrhythmia (RSA) as parasympathetic integrity markers over the sinus node. The RSA is characterized by the physiologic oscillations of HR occurring in synchronicity with the respiratory cycle.^8^,^9^ During respiration, there is an identifiable relationship between inspiration and increased in HR, caused by the withdrawal of vagal tone, and between expiration and reduced in HR, caused by increased vagal activity on the sinus node. An increase in the HR range during the respiratory cycle reflects increased cardiac health.^10^–^11^

COPD has been shown to lead to inspiratory muscle weakness that, in turn, could contribute to a negatively altered cardiovascular response during the respiratory cycle. Previous research has demonstrated that variations in the respiratory rate and tidal volume can modulate the magnitude of respiratory sinus arrhythmia, which is known as an important marker of vagal tone.^10^ However, it is not clear whether the magnitude of sinus arrhythmia is related to respiratory muscle weakness in COPD.

Given this research gap, the objective of the present study was to evaluate the influence of respiratory muscle strength on the magnitude of respiratory sinus arrhythmia. We hypothesized that respiratory muscle weakness negatively influences HR variability during respiratory maneuvers in patients with COPD.

## METHOD

### Study design and patient population

The present study was cross-sectional. The study population comprised 10 males with a clinical diagnosis of COPD according to the Global Initiative for Obstructive Lung Disease criteria (GOLD) and nine age- and gender-matched sedentary control subjects. Inclusion criteria for COPD patients were: forced expiratory volume in one second (FEV1)/forced vital capacity (FVC) ratio < 0.7 and FEV1 < 60% of predicted value; clinical stability for at least three months; nonsmoker status; and presentation of dyspnea under low and medium physical effort. All COPD patients used short-action bronchodilators, and six used long-action bronchodilators. Subjects in the control group were free of chronic pulmonary, cardiovascular, immune, and/or metabolic disease. All participants underwent a clinical evaluation and were screened with pulmonary function tests, functional capacity assessment according to the medical research council (MRC),^13^ analysis of blood biochemistry, electrocardiography, and symptom-limited exercise testing. All participants signed a written indication of informed consent, and the study protocol was approved by the University Ethics Committee.

### Experimental procedure

The volunteers were first familiarized with the experimental room environment and the researchers involved in the study. The COPD patients were instructed to avoid bronchodilators such as B_2_-agonists, xanthene derivates, and steroids for 24 hours before the experimental test. Prior to the initiation of the tests included in the present study, the participants were interviewed (to ensure compliance with medication instructions) and examined. Systolic and diastolic blood pressure, pulmonary auscultation, and peripheral oxygen saturation (SpO_2_) were all assessed. Only one data collection session was required for each subject.

### Measurements

#### Pulmonary Function Test

Spirometric tests were performed using the Vitalograph^®^ spirometer (Hand-Held 2021 instrument. Ennis, Ireland). Slow vital capacity (SVC) and forced vital capacity (FVC) were measured to determine FEV_1_ and the FEV_1_/FVC ratio. Pulmonary function tests were performed in accordance with American Thoracic Society guidelines.^14^

#### Maximal Respiratory Pressures

The MIP was obtained from the residual volume and total lung capacity. Additionally, the maximal expiratory pressure was obtained from a deep inspiration following a maximal expiration. The subjects were seated, wearing nose clips and a mouthpiece. The mouthpiece was connected to a manual shutter apparatus, with maximal pressures measured by an aneroid-gauge manometer (± 300 cmH_2_O) (GERAR, São Paulo, SP, Brazil). Patients were asked to perform maximal inspiratory and expiratory efforts against an obstructed mouthpiece that had a small leak to prevent the patients from closing their glottis during the maneuver. Patients sustained their maximal effort for one second. The best of three consecutive attempts was used to determine MIP, and percent-predicted values were derived.^15^

#### Heart-Rate Variability

First, the volunteers were maintained at rest in the supine position for approximately 10 minutes to ensure that a true resting HR value was obtained. Then, the ECG signal and the instantaneous HR were obtained at rest in the supine position for a duration of 15 minutes. Subsequently, the HR and R-R intervals were recorded during the RSA maneuver (RSA-M) in the supine position in the following order: (1) for one minute at rest with spontaneous breathing; (2) for four minutes while performing the RSA-M; and (3) for one minute at rest with spontaneous breathing. During the RSA-M, the volunteers were instructed to perform a series of deep and slow inspirations and expirations to provide a pulmonary volume that varied from the total lung capacity (maximal inspiration) to the residual volume (maximal expiration). Each respiratory cycle was performed for 10 seconds, i.e., five seconds for inspiration and five seconds for expiration, which corresponds to a breathing rate of six cycles/min. This breathing rate provides a maximal respiratory sinus arrhythmia response, according to Hayano et al.^16^ The participants controlled their breathing rate with a pointer clock, and, at the same time, verbal feedback was provided by the researcher based on observation of the visualized ECG signal and the HR plot on the computer monitor, which confirmed whether or not the respiratory cycle had been performed correctly. Finally, the HR and R-R intervals were recorded at rest in the sitting position for 15 minutes. During all experiments, the subjects were monitored at the CM5 lead. The ECG signal was obtained from a one-channel heart monitor (TC 500, Ecafix, São Paulo, SP, Brazil) and was used to calculate the R-R interval using specific software.^17^

### Heart-Rate Variability Analysis

HRV was analyzed by time and frequency domain methods using an algorithm developed in MatLab (version 6.1, 450 Release 12.1). The section of highest stability, which included a simple line comprised of at least 256 points, was selected from R-R intervals by visual inspection according to the criteria set forth by the Task Force of the European Society of Cardiology and the North American Society of Pacing and Electrophysiology.^18^

Time domain analysis was calculated from the RMSSD index [the square root of the sum of the squares of the differences between adjacent normal-to-normal (NN) intervals] and the SDNN (the standard deviation of NN intervals).^18^ Frequency domain analysis utilized the fast Fourier transform (FFT) of the time series. The application of this algorithm permitted the identification of the power spectral density (PSD) as well as its frequency bands: very low frequency (VLF), low frequency (LF), and high frequency (HF). Two frequency bands that best represent the vagal and sympathetic activity of HR control were used in this study. Signals in the LF band (0.04 to 0.15 Hz) have been predominately attributed to high sympathetic tone and low parasympathetic tone. On the other hand, signals in the HF band (0.15 to 0.4) have been attributed only to parasympathetic activity.^18^ Spectral components were obtained in both absolute (ms^2^) and normalized units (nu).^18^

Time and frequency analysis of the R-R intervals acquired during RSA-M was then performed. Additionally, the spectral analysis confirmed that all volunteers maintained a respiratory rate between five and six cycles/min, which corresponds to a peak spectral density frequency between 0.08 to 0.1 Hz. Another specific routine developed in MatLab was used to calculate the HR and R-R interval indices of RSA-M,^20^ i.e., the expiratory/inspiratory ratio (E/I), which is the mean of the longest R-R interval values obtained during the expiratory phase divided by the mean of the shortest R-R interval values obtained during the inspiratory phase, and the inspiratory-expiratory difference (ΔIE), which is the difference between the mean of the highest HR value obtained during the inspiratory phase and the mean of the lowest HR value obtained during the expiratory phase.

Finally, the data obtained by HRV analysis in the time and frequency domains as well as the HR and R-R interval indices of RSA-M were transformed into decimal logarithms for statistical analysis.

### Statistical Analysis

The sample size was calculated using GraphPad StatMate software, version 1.01. To reach statistical significance (p<0.05 at a power of 90% with a confidence interval of 95%), a sample of nine subjects in each of the groups was needed to show differences in the physiologic variables (ΔIE and SpO_2_). Due to the normal distribution (Shapiro-Wilk test) and to the variance homogeneity (Levene Test) of the data, parametric tests were selected for statistical analysis. For the inter-group comparison (COPD Group vs. Control Group), unpaired Student’s t-test was applied. The Pearson correlation was used to observe the inter-variable relationship. All statistical analyses were carried out using SPSS version 10 (Chicago, IL) with the level of significance set at p<0.05. All data are expressed as means and standard deviations.

## RESULTS

### Subject characteristics

The subject characteristics are presented in Table 1. There were no differences in age, height, or body mass index (BMI) between the groups. However, COPD patients presented lower body weight when compared to the control group. In addition, the COPD group was classified as GOLD values of the COPD Stage IIb. Spirometric results and SpO_2_ group were significantly lower than those of the healthy individuals were. The COPD group was classified as MRC I (n=1), II (n=3), and III (n=6). As expected, MIP was significantly reduced in the COPD group in comparison to the control group (p<0.001).

### Cardiac autonomic control at rest

**Figure 1:**
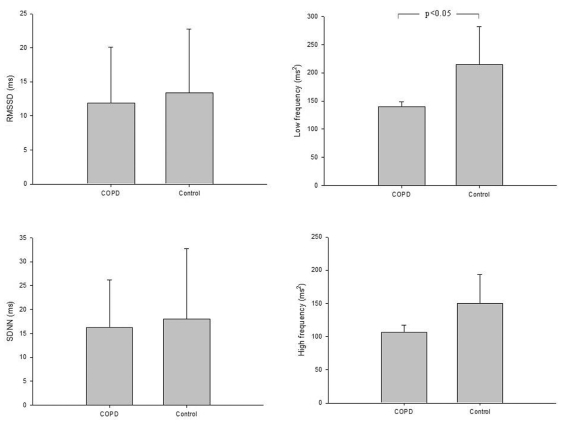
Comparison of the heart-rate variability index obtained at rest for all groups. (A) RMSSD: the square root of the sum of the squares of differences between adjacent NN intervals; (B) SDNN: the standard deviation of NN intervals; (C) High frequency; and (D) Low Frequency.

The COPD patients presented a significant reduction in LF in comparison to the control group (p<0.05) (Figure 1). However, RMSSD and SDNN indices and HF did not present significant differences between the groups.

### Cardiac autonomic control during RSA-M

**Figure 2:**
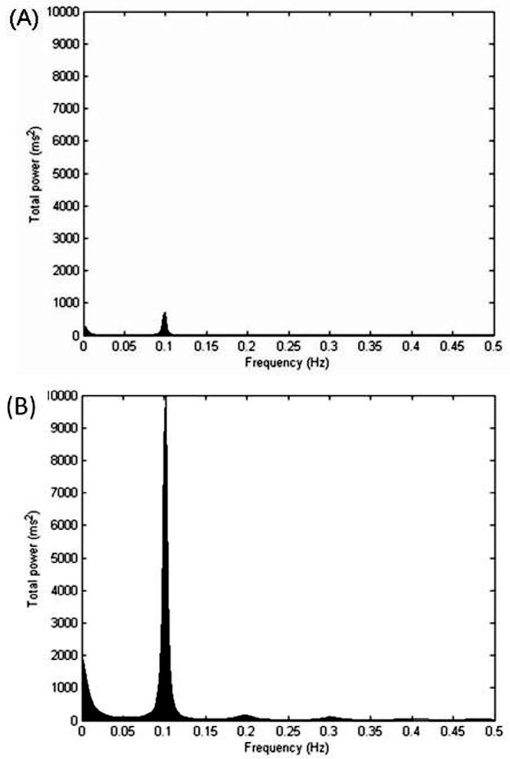
Decomposition of the spectrum into single spectral components of very low frequency (VLF), low frequency (LF), and high frequency (HF) during the respiratory sinusal arrhythmia maneuver. (A) COPD patients; (B) Control group.

Table 2 shows the time domain analysis of HRV during RSA-M. The COPD group showed lower values for the E/I ratio and ΔIE when compared to the control group (p<0.05). Additionally, lower values for the RMSSD and SDNN indices were observed in COPD in comparison to the control group. Figure 2 illustrates examples of the entire HRV power spectrum broken down into single spectral components from representative patients in the COPD and control groups. The representative COPD patient presented lower spectral components compared to the control (p<0.05).

### Relationships between maximal inspiratory pressure and cardiac autonomic control

**Figure 3:**
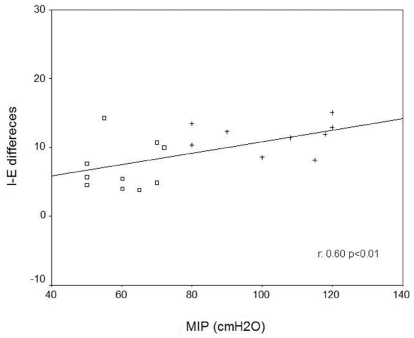
Pearson correlation. Correlation between MIP and inspiratory-expiratory differences (I-E differences). (Legend: □ = COPD and + = Control).

Figure 3 shows the relationship between MIP and HRV parameters. There was a significant positive relationship between MIP and the ΔIE (r = 0.60, p<0.01).

## DISCUSSION

The primary findings of the present study were as follows: 1) COPD patients showed evidence of impaired autonomic modulation of heart rate at rest and during RSA-M; 2) the relationship between the MIP and HRV indices during RSA-M indicates that the inspiratory muscle weakness observed in this population may be associated with cardiac autonomic control.

### The HRV at rest

It has been observed in many chronic disease populations that the sympathetic-vagal balance of the sinus node is impaired, favoring sympathetic tone. In COPD, the coexistence of airflow limitation and the loss of the lungs’ elastic recoil results in modifications to the breathing pattern and in the volume and capacity of the lungs. For this reason, a series of adjustments through the interaction between the peripheral-central receptors and the cardiopulmonary control center, especially in the autonomic nervous system, occur to guarantee the body’s homeostasis.^21^

In the present study, the COPD group showed a greater reduction in LF values compared to the control group. However, the cardiac autonomic control in patients with COPD is unclear. Some authors^22^–^24^ have shown that these patients present a reduction in HRV but a predominance of parasympathetic activity when compared to healthy individuals. It is thought that accentuated vagal effects on cardiac autonomic control reflect vagal hyperactivity in the airway. On the other hand, Stewart et al.^25^ and Chen et al.^26^ found significant sympathetic activity in COPD patients. The other plausible explanation in patients with COPD is a chemoreflexive activation of sympathetic outflow. These patients could present chronic hypoxemia of peripheral tissue, which can modify autonomic control via peripheral and central chemoreceptors.^4^,^27^ We speculated that these autonomic control derangements in COPD patients could be related to the inflammatory stress and peripheral and respiratory muscle dysfunction present in this disease.

### The HRV during RSA-M

The HR and R-R interval indices obtained during the RSA-M provided relevant information concerning the vagal activity at the sinus node in these patients. The COPD patients showed a reduced parasympathetic response when compared to the matched control group. The results of the present study reaffirm previous findings demonstrating significant RSA-M reduction in COPD^28^,^29^ patients when compared to healthy volunteers. This sinus arrhythmia is modulated by the interaction between the cardiovascular and pulmonary systems. In this context, alterations in respiratory frequency and tidal volume can alter this relationship.^7^ Because the respiratory rate was controlled during RSA-M, we believe that the lower RSA-M values might result from the reduction in tidal volume. Altered pulmonary compliance may be a primary mechanism for this phenomenon. As a result of hyperinflation, the residual volume may be increased in patients with COPD, thus decreasing the range of the tidal volume variation. Thus, although during the RSA-M the volunteers were instructed to perform a series of deep and slow inspirations and expirations, the tidal volume mobilized could have been minimal due to the increased residual volume.

### Respiratory muscle weakness and its relationship to cardiac autonomic control

Our evaluation of the relationships between MIP and RSA-M indices and between MIP and HRV indices indicated that subjects with respiratory muscle weakness exhibited a more pronounced reduction of vagal tone at the sinus node. Therefore, alterations of respiratory parameters can profoundly influence RSA-M magnitude and HRV behavior; slow and deep breathing will amplify RSA-M magnitude and HRV behavior, whereas fast and shallow breathing may contribute to reduced RSA-M magnitude and HRV behavior.^7^,^10^ Assuming that respiratory muscle weakness leads to shallow breathing and considering that normal incursion was shown to be limited by the diaphragm, the ergoreceptor may be activated early and may consequently be responsible for the fast central response in cardiac autonomic control.

Recent studies have shown that respiratory muscle strength could affect the HRV and the RSA-M magnitude in COPD patients with respiratory muscle weakness.^30^ Therefore, this method of evaluation may be important for evaluating the alteration of cardiac autonomic control mediated by the ergoreflex in the central nervous system’s cardio-respiratory center, although the precise mechanism remains unclear. In conclusion, COPD is characterized by impairment of the sympathetic-vagal balance at rest. These alterations in cardiac autonomic control of HR are associated with inspiratory muscle weakness in these patients and bring to light a potentially valuable target for rehabilitation.

### Limitations

The present study does possess limitations. First, measurement and control of the tidal volume during respiratory control, which was not carried out in this study, could have contributed to the consolidation and interpretation of the results found herein. Second, the evaluation of complete lung function (static volumes) would be especially relevant when evaluating these patient populations. These additional measurements would have involved the use of prohibitively expensive equipment and could therefore not be included.

### Clinical implications

These results could be important for identifying maladjustment in cardiac modulation and suggest the importance of rehabilitative strategies such as respiratory muscle training for patients with COPD who present significant respiratory muscle weakness. It is well documented that respiratory muscle training significantly improves pulmonary function in patients with COPD.^31^,^32^

It is presently unknown whether an improvement in respiratory function equates to improved cardiac autonomic tone in these patient populations.^43^ Demonstration of improved autonomic tone through respiratory muscle training would further bolster support for the implementation of this therapeutic intervention. Future research is required to clarify these issues.

## Figures and Tables

**Table 1:** Demographic, anthropometric, and clinical characteristics for all groups.

Variables	COPD (N = 10)	Control (N = 9)
***Demographics / Anthropometrics***		
Age (yrs)	69 ± 9	64 ± 5
Height (m)	1.67 ± 0.08	1.71 ± 0.05
Weight (kg)	64 ± 9.0*	75 ± 6.3
BMI (kg/m^2^)	23 ± 3.3	25 ± 1.2
***Spirometrics***		
FEV_1_ (% predict)	41 ± 11*	91 ± 20
FVC (% predict)	68 ± 13*	102 ± 15
FEV_1_/FVC	59 ± 12*	101 ± 7
***Clinical characteristics***		
MIP (cmH_2_O)	60 ± 9*	103 ± 15
MIP (% predict)	63 ± 11	99 ± 17
SpO_2_ (%)	92 ± 3*	96 ± 1
RF (bpm)	15 ± 4	12 ± 3

Values are means ± SD. COPD: chronic obstructive pulmonary disease; BMI: body mass index; EF: left ventricle ejection fraction; FEV_1_: forced expiratory volume in the first second; FEV_1_/FVC: forced expiratory volume in the first second to forced vital capacity ratio; MIP: maximal inspiratory pressure; SpO_2_: peripheral oxygen saturation; RF: respiratory frequency;

* p<0.05: COPD vs. Control (unpaired Student’s t-test).

**Table 2:** I/E ratio, IE differences, and heart-rate variability during respiratory sinusal arrhythmia maneuver for the studied groups.

	COPD (n = 10)	Control (n = 9)
***Time domain***		
E/I ratio	1.1 ± 0.06	1.2 ± 0.1*
ΔIE	7.0 ± 3.5	12.7 ± 4.2*
RMSSD	18.3 ± 15.6	43.5 ± 27.9*
SDNN	32.1 ± 21.2	63.8 ± 29.1*
***Frequency domain***		
LFab	1052.7 ± 1538.3	3551.2 ± 3581.2*
HFab	93.9 ± 153.8	626.8 ± 906.8*
LFun	0.9 ± 0.03	0.8 ± 0.07
HFun	0.1 ± 0.03	0.2 ± 0.07
LF/HF	15.9 ± 15.3	11.3 ± 7.5

Values are means ± SD. COPD: chronic obstructive pulmonary disease; E/I ratio: expiratory/inspiratory ratio; ΔIE: inspiratory-expiratory differences; RMSSD: the square root of the sum of the squares of differences between adjacent NN intervals; SDNN: the standard deviation of NN intervals; LFab: low frequency in absolutes values; HFab: high frequency in absolutes values; LFun: low frequency in normalized units; HFun: high frequency in normalized units;

* p<0.05: COPD vs. Control (unpaired Student’s t-test).
